# Machine Learning Applications in Solid Organ Transplantation and Related Complications

**DOI:** 10.3389/fimmu.2021.739728

**Published:** 2021-09-16

**Authors:** Jeremy A. Balch, Daniel Delitto, Patrick J. Tighe, Ali Zarrinpar, Philip A. Efron, Parisa Rashidi, Gilbert R. Upchurch, Azra Bihorac, Tyler J. Loftus

**Affiliations:** ^1^Department of Surgery, University of Florida Health, Gainesville, FL, United States; ^2^Department of Surgery, Johns Hopkins University, Baltimore, MD, United States; ^3^Department of Anesthesiology, University of Florida Health, Gainesville, FL, United States; ^4^Department of Orthopedics, University of Florida Health, Gainesville, FL, United States; ^5^Department of Information Systems/Operations Management, University of Florida Health, Gainesville, FL, United States; ^6^Department of Biomedical Engineering, University of Florida, Gainesville, FL, United States; ^7^Department of Computer and Information Science and Engineering University of Florida, Gainesville, FL, United States; ^8^Department of Electrical and Computer Engineering, University of Florida, Gainesville, FL, United States; ^9^Precision and Intelligent Systems in Medicine (PrismaP), University of Florida, Gainesville, FL, United States; ^10^Department of Medicine, University of Florida Health, Gainesville, FL, United States

**Keywords:** machine learning, transplantation, artificial intelligence, organ allocation, critical care

## Abstract

The complexity of transplant medicine pushes the boundaries of innate, human reasoning. From networks of immune modulators to dynamic pharmacokinetics to variable postoperative graft survival to equitable allocation of scarce organs, machine learning promises to inform clinical decision making by deciphering prodigious amounts of available data. This paper reviews current research describing how algorithms have the potential to augment clinical practice in solid organ transplantation. We provide a general introduction to different machine learning techniques, describing their strengths, limitations, and barriers to clinical implementation. We summarize emerging evidence that recent advances that allow machine learning algorithms to predict acute post-surgical and long-term outcomes, classify biopsy and radiographic data, augment pharmacologic decision making, and accurately represent the complexity of host immune response. Yet, many of these applications exist in pre-clinical form only, supported primarily by evidence of single-center, retrospective studies. Prospective investigation of these technologies has the potential to unlock the potential of machine learning to augment solid organ transplantation clinical care and health care delivery systems.

## Introduction

The inherent complexity and rapid, multidisciplinary growth of transplant medicine stands to benefit from new tools to inform clinical decision making. Machine learning offers the promise of rendering troves of routinely collected data actionable in the field of transplantation.

Machine learning is a branch of artificial intelligence in which a computer algorithm learns from examples to generate reproducible predictions and classifications on previously unseen data (1, 2). Machine learning can be supervised or unsupervised: the former referring to manually mapping an observation’s characteristics to a known outcome; the latter referring to discovery of innate patterns using unlabeled data (1). An example of supervised learning would include using known clinical risk factors to predict survival. In contrast, an unsupervised model could be fed thousands of histopathology slides and learn to group them according to similarities in pixel patterns. A further subset of machine learning includes neural networks. These networks rely on layers of calculation-performing nodes that differentially weight inputs before passing them along to other nodes, eventually producing a narrow range of outputs. Deep neural networks consist of dozens to hundreds of layers, often with specialized functions interspersed within the layers, enabling the network to better represent complex patterns in unstructured data.

**Figure 1:**
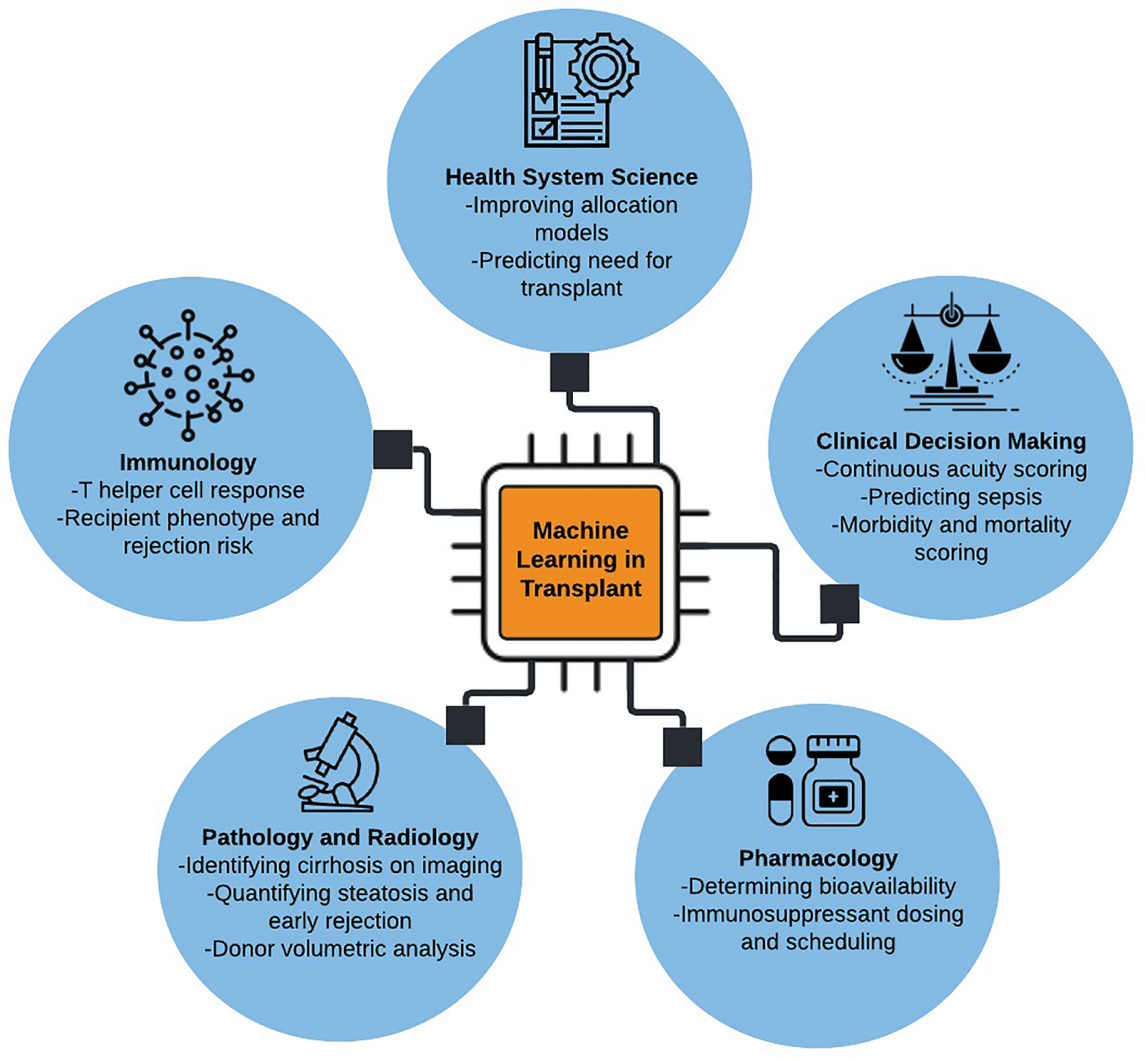
Illustrative summary of machine learning applications in solid organ transplantation.

Several excellent reviews have been published on machine learning in transplant medicine (3–6). This review builds on prior knowledge by incorporating additional applications of machine learning in predicting acute post-surgical and long-term outcomes, caring for critically ill patients, classifying biopsy and radiographic data, augmenting pharmacologic decision making, and elucidating the complexity of host immune response. A glossary of machine learning terms and applications in solid organ transplantation is provided in **Table 1**. Major applications of machine learning in solid organ transplantation are illustrated in **Figure 1**.

**Table 1:** A glossary of machine learning terms and applications in solid organ transplantation.

Term	Definition	
Machine Learning	A sub-field of artificial intelligence in which a computer system performs a task without explicit instructions	
Deep Neural Networks	A sub-field of machine learning in which computer systems learn and represent data by adjusting weighted associations among input features across a layered hierarchy of neurons or neural network	
Supervised Learning	Algorithms learn from training sets of labeled data and then classifies new, previously unseen data	
Unsupervised Learning	Algorithms learn from unlabeled data and generate their own classification schemes, which can discover hidden patterns	
**Term**	**Definition**	**Application in Transplant Medicine**
Clustering Analysis	Arranging data objects into groups based on similarities among objects	Phenotyping kidney transplant recipients with highest risk of rejection (7)
Convolutional Neural Networks	Neural networks that represent patterns in two-dimensional data, used frequently in image processing	Ultrasound to identify cirrhosis (8) Quantifying hepatic steatosis in liver biopsies (9) Early assessment of transplanted kidney function on biopsy (10) Predicting critical illness from EMR data (11) Liver segmentation and volumetric analysis of living donors (12)
Ensemble learning	Combines multiple decision trees into one model, capitalizing on the individual strengths and weaknesses of individual predictions	Identifying modifiable risk factors for mortality in liver transplant recipients with diabetes (13) Predicating acute kidney injury after liver transplant (14) Estimating bioavailability of tacrolimus in renal transplant (15) Predicting mortality for liver transplant candidates (16, 17) Optimizing tacrolimus dosing in renal transplant recipients (18)
Gated Recurrent Units	Specify how information is stored and filtered in a recurrent neural network	Predicting sepsis in ICU patients (19) Continuous acuity scoring for ICU patients (20)
Modified U-net model	Type of convolutional neural network that can use smaller training sets with greater output resolution	Quantifying hepatic steatosis in liver biopsies (21)
Random Forest Model	Multiple, uncorrelated decision trees whose accuracy is greater than the sum of individual trees	Predicting survival after liver transplantation (22) Predicting liver graft failure (23) Predicting acute kidney injury after liver transplantation (14)
Recurrent Neural Networks	Neural networks that remember past decisions and can process data in temporal sequence, i.e. time series data	Predicting risk for sepsis (19) Predicting in-hospital mortality (20)
Reduced Error Pruning Tree	Elimination of redundant classification trees to reduce overfitting	Identifying hepatitis C virus genotypes associated with advanced fibrosis (24)
Reinforcement Learning	Optimizes the probability of achieving an objective in a particular situation or environment	T helper cell response to effector molecules (25) Optimizing clinical decision making in treating sepsis (26)
Support Vector Machine	Defines a plane that optimally separates two classes of data points	Predicting acute kidney injury after liver transplant (14) Predicting bronchiolitis obliterans with CT imaging (27)

ICU, intensive care unit.

## Predicting Clinical Outcomes After Solid Organ Transplantation With Machine Learning

Accurate prognostic information is essential for informed decision-making in transplant medicine, both at bedside and in allocation policy. Current models—including the Donor Risk Index, Model for End-Stage Liver Disease (MELD), and the Survival Outcomes Following Liver Transplantation (SOFT) scores—offer moderate success in predicting who would benefit from transplant; yet these models are not without their critics. MELD requires exception points for certain medical conditions and may disproportionately allocate organs to patients with medically stable hepatocellular carcinoma (28). Donor Risk Index has limited generalizability, relies on variable ratings of pathology, and has had limited clinical adoption (23). The SOFT score and its variations, while accurate in predicting mortality, are less successful in predicting morbidity (29). Most survivorship models in heart, liver, kidney, and lung transplant assume linear relationships between non-interacting input variables and mortality; and their relationship is often summarized with Cox proportional hazards models. While offering a straightforward interpretation through hazard ratios, they are limited both by the number of variables that can be compared and by the linear relationship among those variables. Machine learning and neural networks offer the synthesis of non-linear associations among a greater number of inputs.

Multiple models have been proposed in recent years. Risk features for acute, postoperative outcomes, including mortality and acute kidney injury, were identified using a gradient boosting machine (13, 14). Molinari et al. (17) bridged traditional methods by developing a point-system for predicting 90-day mortality in liver transplant using variables identified through artificial neural networks, classification tree analysis, and logistic regression. Lau et al. (23) compared Donor Risk Index and Survival Outcomes following Liver Transplantation scores using artificial neural networks and random forest models to identify 15 important factors and were able to improve upon the pre-existing models (AUC-ROC value from 0.680 to 0.818). Yoon et al. (30) generated a tree of predictors for cardiac transplant and Kantidakis et al. (22) demonstrated improved predictability using random forest survival analysis in kidney transplant compared with Cox models. Bertsimas et al. (16) compared an optimal classification of trees model to MELD scoring, demonstrating that the machine learning approach had both improved accuracy and the potential to decrease mortality from sub-optimal organ allocation.

Specific genotypes can place individuals at higher risk of need for liver transplantation. Shousha et al. (24) constructed a specialized data mining analysis using a reduced error pruning tree to identify variables associated with liver fibrosis in patients infected with hepatitis C. They found that the genotype IL28B were most heavily weighted by the algorithm when predicting advanced fibrosis. Barriers exist to the adoption of these models. The algorithms may be trained on single-institution datasets, thus lacking generalizability. They also suffer from overfitting, lack of integration into pre-existing EMRs, involvement of proprietary software that limits adoptability, or inadequate interpretability. At its most basic level, machine learning may not offer improvement over currently existing statistical methods. Kantidakis et al. (22) demonstrated only a marginal improvement in IBS and C-index for predicting survival in kidney transplants with their advanced computational models; Miller et al. (31) demonstrated no added benefit of their models to current risk indices.

Organ scarcity, healthcare resource intensity, and patient goals of care mandate both subjective and objective assessments of transplant candidacy. Predicted patient survivorship and outcomes are an inseparable part of these discussions; machine learning may be able to offer more equitable estimates–and possible inclusion of alternative outcomes–to guide both individual and system-wide organ allocation. Several authors have validated their work on national datasets (16, 17, 22, 31) and would benefit from trials of integration of these models into electronic medical records to help guide the physician-patient discussion. Eventual changes to national allocation algorithms would require both further validation and weighting of stake holder realities.

## Aligning Patient Acuity With Resource Intensity After Solid Organ Transplantation

Clinical outcomes are highly dependent on the timely recognition of complications in the immediate postoperative period. Protocols for posttransplant management vary by institutional, surgeon, and patient factors. Cardiac, lung, and liver transplant recipients almost universally require admission to a surgical intensive care unit following their procedure, though duration of stay does not necessarily reflect patient acuity. Similarly, predicting decompensation on surgical wards can help avoid failure-to-rescue by pre-emptively triaging patients back to intensive care units.

Research into machine learning has touched upon identifying patients at high risk for decompensation. Lee et al. (14) were able to predict onset of acute kidney injury following liver transplant using a support vector machine and random tree analysis. More generally, DeepSOFA and Artificial Intelligence Sepsis Expert (AISE) were able to provide real-time acuity predictions for patients at risk for sepsis that outperformed SOFA and APACHE models using recurrent neural networks, grated recurrent units, and modified Weibull-Cox proportional hazards models (19, 20). A reinforcement learning algorithm outperformed clinicians in determining theoretical ideal fluid resuscitation and vasopressor use (26).

Outputs are often meaningless to clinicians without a clear path to how they were obtained. Shickel et al. (20) and Lauristen et al. (11) were able to create interpretable models by reversing the algorithm to highlight which inputs weighed most heavily in deciding the outputs. A variety of tools exist explicitly for this purpose.

While many of these tools have yet to be validated in a transplant cohort, they have potential applicability for identifying decompensation or acute rejection. Recording of continuous, multivariate physiologic input features and real-time integration into the EHR could improve these models’ predictive abilities along with their usability to clinicians.

## Classifying Solid Organ Biopsy Specimens and Radiographic Findings With Computer Vision

Since the early 1980s, computers have assisted in radiographic interpretation. Evolving from low-level pixel processing and image editing to segmentation and feature extraction and recently to the introduction of convolutional neural networks, imaging analytics have been at the forefront of machine learning in medicine (32). Multiple studies highlight the inter- and intra-user variability in interpreting both diagnostic imaging and biopsy specimens (33–35). This can have profound implications when assessing organ injury, donor organ viability, and acceptance of newly transplanted organs. There are practical implications too, with the off-hours timing of organ procurement, transplant, and complications, when accurate diagnosis is time sensitive.

Esteva et al. (36) demonstrated that deep neural networks can outperform dermatologists in classifying images of skin lesions. Computer vision, or the field of teaching computers to “see”, takes advantage of convolutional neural networks (CNN) to take clusters of pixels and employ a weighted pattern recognition to classify images into groups that can correspond to disease vs non-diseased states. Prior to transplant, Liu et al. (8) were able to combine the descriptive power of CNN with the generalizable features of support vector machines to produce a model that accurately identifies cirrhosis on ultrasound. Liu’s model was trained on only 91 images, suggesting that the same approach can be applied to rare conditions for which gold standard images are sparse. Models can also take both supervised and unsupervised approaches. Kuvar et al. (12) compared semi-automatic (requiring human input) with fully automatic algorithms for liver segmentation and volumetric analysis in living liver donors. The latter had improved functionality without the requirement of user interactions, though it does require enormous amounts of memory and processing power.

At time of procurement, donor liver viability can be impaired by steatosis, requiring accurate and timely assessment of biopsy specimens. Reliable analysis is limited given the variations in size, shape, overlap, and clumping of adipose droplets. Roy et al. (21) were able to create an unsupervised, modified U-net model that distinguished true droplets, providing correlations with pathologist measurements, radiology readouts, and clinical data. Sun et al. (9) developed a similar model that consistently out-performed the on call pathologist. Both models used VGG16 (Visual Geometry Group, Oxford), a computer vision CNN trained on millions of random images and modified its uppermost classification layers to identify steatosis.

Post-transplant complications are often diagnosed on biopsy, though integration of machine learning into imaging may obviate this need and its attendant risk for hemorrhage, infection, and damage to adjacent anatomical structures. Diffusion weighted MRI images have been added to models of non-imaging inputs, including creatine clearance and serum plasma creatine, to reveal early graft dysfunction at greater than 90% sensitivity and specificity (10). Late graft dysfunction, including bronchiolitis obliterans after lung transplant, can be seen early with computed tomography scans and functional respiratory imaging (27).

When biopsy is required, tissue molecular analysis may augment visual assessment. Reeve et al. (7, 37) used a clustering analysis to group phenotypes associated with kidney transplant rejection. The algorithm–trained on 1,208 kidney biopsies–sought to replace empiric, yes/no assessments of indeterminant histologic diagnosis of rejection with a probabilistic model using algorithm-identified subtypes of T cell- (TCMR) and antibody-mediated rejection (ABMR). Liu et al. (38) used the statistical method of linear discriminant analysis combined with machine learning techniques to demonstrate the feasibility of using mRNA to diagnosis TCMR in biopsy specimens. These studies were furthered and commercialized by Halloran et al. (39) in the development of the Molecular Microscope^®^ Diagnostic System (MMDx). Using support vector machines, principal component analyses, and classification trees to parse out mRNA expression variation in kidney transplant biopsies, this technology hopes to compliment, if not surpass, histology in determining rejection phenotype.

Machine learning promises to augment clinical decisions shared by transplant patients and practitioners. While the need for human supervision and ultimate responsibility remains strong, these tools have the stated goals of reducing intra- and inter-user variability and providing baseline risk profiles for early disease and organ assessment in both the donor and recipient.

## Machine Learning for Personalized Pharmacokinetics in Solid Organ Transplantation

One of the major challenges in managing solid organ transplant is determining optimal dosing of immunosuppression and other dose-sensitive medications. This task is especially challenging in the context of renal and hepatic dysfunction. Waiting for stabilization of drug levels can result not only in delay of discharge, but also graft failure and adverse drug events. Falling into the realm of personalized medicine, patient specific phenotypes play an important role in pharmacokinetics, suggesting utility for incorporating pharmacokinetic data into machine-learning algorithms to improve clinical dosing decisions.

Two studies examined artificial intelligence in predicting tacrolimus bioavailability and dosing. Using input features of creatinine, body mass index, age, sex, ABCB1 polymorphisms, and CYP3A5^∗^3 genotypes, Thishya et al. (15) developed a three-level artificial neural network to show which variables increased tacrolimus bioavailability and risk of diabetes in renal transplant recipients. Also in renal transplant recipients, Tang et al. (18) compared linear regression to eight machine learning models to predict stable tacrolimus dosing. Regression trees had the best overall performance, however, while superior to linear regression, their predictive ability was similar to the seven other machine learning techniques.

The question remains as to what clinical benefit these machine learning models provide. Less cumbersome mathematical models have demonstrated efficacy in optimizing medication dosing. Zarrinpar et al. (40) developed a parabolic personalized dosing model based on a second order polynomial equation that consistently resulted in appropriate tacrolimus dosing changes in liver transplant recipients. Current dosing strategies are largely based on physician and pharmacologist experience. Future tools will need to incorporate patient level factors to build more objective and consistent dosing schemes.

## Complexity of the Host Immune Response to Transplantation and Immunosuppression

The human immune system is too complex to be interpreted by additive or linear models alone. Within transplant medicine, immunosuppression adds additional layers of complexity that are often beyond our innate reasoning’s ability to interpret, and thus requiring computational assistance.

As early as 1970, the integration of computer science into medicine was predicted to be inevitable (41). The rapid expansion of medical knowledge included insights to our own limits. In diagnosis, we are hampered by our inability to intuitively multiply probabilities; and our decision making is informed more by heuristics–also called mental shortcuts–than assimilation of all available data (29). Innate and adaptive immunity is influenced by environmental cues regarding the presence of pathogens, subtle structural changes in ligand-receptor complexes, circulating stimulatory and inhibitory mediators, and a host of other complex processes. Transplant immunology further complicates this system by introducing foreign antigens and relatively nonspecific immune targeting agents. The interplay between all these factors is far too complex to model by adding the individual parts together, particularly since our knowledge of each part is itself quite limited.

Growth in the field of reinforcement learning has informed our understanding of many biologic systems, though its application to the immune system is limited (42). Kato et al. created a theoretical framework using reinforcement learning and reward systems to understand how T helper cells bias the responses of their effectors towards elimination of pathogens (25). With greater than 10 (16) unique receptor sequences and 10 (28) unique T cells, these models are essential given the shear diversity of adaptive immune cells (43).

Publications at the intersection of transplant immunology and machine learning are, however, limited. As we have seen, there is potential in adding genotypic and cell-expression variables into models predicting both need for transplant and risk of graft failure.

## Limitations

While limitations specific to individual studies were presented above, it is worth restating and consolidating the limits of these technologies more generally. Most studied algorithms are trained on single-institution, proprietary datasets. This may provide improved quality of data and inclusion of more specific variables but can result in overfitting, or where the model incorporates the irrelevant noise into its final predictions and cannot be generalized onto broader data sets. This is notable when studies fail to mention external validation of their model. Secondly, machine learning models may fail to offer improvement over current models. As mentioned above, multiple studies have no additional benefit in predictive ability despite the extra computing power (22, 31, 40).

More problematic is the lack of studies failing to demonstrate clinic relevance. The use of machine learning in medicine in still young and research is often focused on the predictive abilities rather than improvement in patient outcomes. Computer-guided decision making and its impact on graft survival, surgical complications, or mortality are difficult to undertake and may face further practical barriers in obtaining approval of Institution Review Boards.

Once these limitations are addressed, integration into workflow and clinical practice remain a perennial problem. There are many opportunities for inclusion of decision-making tools into the EHR are available, but alarm fatigue, ease of use, cost to implementation, value to patients and providers, and even mistrust of these services will hinder their adoption.

## Conclusions

Machine learning offers the potential to synthesize large quantities of routinely collected clinical data into clinically applicable recommendations. Recent advances allow machine learning algorithms to accurately represent the complexity of host immune response, predict acute post-surgical and long-term outcomes, classify biopsy and radiographic data, and augment pharmacologic decision making. Yet, many of these applications exist in pre-clinical form only, supported primarily by evidence of single-center, retrospective studies. Prospective investigation of these technologies has the potential to unlock the potential of machine learning to augment solid organ transplantation clinical care and health care delivery systems.

## Author Contributions

JB – Literature review, drafting, editing. DD – Literature review, drafting, editing. PT – Editing, subject matter expert. AZ – Editing, subject matter expert. PE – Editing, subject matter expert. PR – Editing, subject matter expert. GU – Editing, subject matter expert. AB – Editing, subject matter expert. TL – Drafting, editing, subject matter expert. All authors contributed to the article and approved the submitted version.

## Funding

PT was supported by R01GM114290 from the NIGMS and R01AG121647 from the National Institute on Aging (NIA). PR was supported by National Science Foundation CAREER award 1750192, P30AG028740 and R01AG05533 from the NIA, 1R21EB027344 from the National Institute of Biomedical Imaging and Bioengineering (NIBIB), and R01GM-110240 from the NIGMS. AB was supported by R01GM110240 from the NIGMS and 1R21EB027344 from the NIBIB. TJL was supported by the National Institute of General Medical Sciences of the National Institutes of Health under Award Number K23 GM140268.

## Author Disclaimer

The content is solely the responsibility of the authors and does not necessarily represent the official views of the National Institutes of Health.

## Conflict of Interest

The authors declare that the research was conducted in the absence of any commercial or financial relationships that could be construed as a potential conflict of interest.

## Publisher’s Note

All claims expressed in this article are solely those of the authors and do not necessarily represent those of their affiliated organizations, or those of the publisher, the editors and the reviewers. Any product that may be evaluated in this article, or claim that may be made by its manufacturer, is not guaranteed or endorsed by the publisher.

## Abbreviations

APACHE, Acute Physiology And Chronic Health Evaluation; AUC-ROC, Area Under the Curve – Receiver Operating Curve; AISE, Artificial Intelligence Sepsis Expert; CNN, Convolutional Neural Network; EMR, Electronic Medical Record; MELD, Model for End-Stage Liver Disease; SOFT, Survival Outcomes Following Liver Transplantation.
